# Wound care antiseptics - performance differences against *Staphylococcus aureus* in biofilm

**DOI:** 10.1186/s13028-015-0111-5

**Published:** 2015-05-04

**Authors:** Lene K Vestby, Live L Nesse

**Affiliations:** Department of Laboratory Services, Section for Bacteriology - Aquatic and Terrestrial, Norwegian Veterinary Institute, Ullevålsveien 68, Pb 750 Sentrum, N-0106 Oslo, Norway

**Keywords:** Biofilm, *Staphylococcus*, Antiseptics, Wounds, Biguanide, QAC, Iodine

## Abstract

**Background:**

*Staphylococcus aureus* is commonly isolated from infected wounds both in animals and humans. It is known to be an excellent biofilm former and biofilms are present in as many as 60% of chronic wounds. Despite that the presence of biofilms in infections are common, antiseptics are usually qualified for *in vivo* testing according to their effect on planktonic cells. As it is well known that bacteria in biofilms are more tolerant to antiseptics than planktonic bacteria, biofilm infections can be difficult to treat. The aim of the study was to compare three different categories of antiseptics, biguanide (chlorhexidine), quaternary ammonium compound (QAC; Pyrisept) and iodine/iodophores (2% iodine liniment), with regards to efficacy in killing *S. aureus* in biofilm. If there was observed a difference in efficacy between these antiseptics, a second aim was to find the most effective of the three antiseptics.

**Results:**

Large differences in the bactericidal effect of the different antiseptics against *S. aureus* in biofilm were observed in the present study. Iodine treatment was found to be the most effective followed by Pyrisept and chlorhexidine.

**Conclusions:**

The bactericidal effect of the different antiseptics used in the present study was found to vary significantly against *S. aureus* in biofilm. The present study gives valuable knowledge with regards to selecting the antiseptics that are most likely to be successful in treating biofilm infected wounds. This study also contributes to focus attention on the importance of qualifying antiseptics based on results using biofilm bacteria rather than planktonic bacteria.

## Background

In most natural environments, including in clinical infections, bacteria form biofilm by encasing themselves in a self-produced extracellular protective matrix [1,2]. The understanding of the clinical importance of biofilm in wounds is relatively new. By using advanced microscopy it was demonstrated that as many as 30 of 50 chronic wounds harbored biofilms [3]. Chronic wounds can be colonized with several different microorganisms of which *Staphylococcus aureus* is the most commonly isolated bacterial species [4-6]. *S. aureus* is known to be an excellent biofilm former and is also found to be able to form biofilm in wounds [3].

A biocide is a general term of describing chemical agents that inactivate microorganisms. Antiseptics are defined as biocides that either destroy or inhibit the growth of microorganisms in or on living tissue. Antiseptics are generally qualified for *in vivo* testing according to their effect on planktonic bacteria although it is well known that bacteria in biofilms are more tolerant to antiseptics than planktonic bacteria, thus making infections harboring biofilms difficult to treat [2]. Several different categories of antiseptics are in use in wound care, including biguanide, quaternary ammonium compounds (QACs) and iodine/iodophores [7]. The working actions of antiseptics are different and for antiseptics like iodine/iodophores, the exact mode of action is still unknown [7]. For other antiseptics like QACs and biguanides, the modes of action are more documented. In general, QACs acts by binding to the bacterial cell membrane with the cationic group facing outwards. The hydrophobic tails are inserted into the lipid bilayer and cause disruption and leakage of cellular content [7,8]. Biguanides have a working action by absorption to the cell membrane by electrostatic interaction [9].

In the present study, three different non-prescriptive antiseptics that are commonly used in animal and human wound care were tested against *S. aureus* in biofilm. The aim of the study was to compare these three antiseptics with regards to efficacy in killing *S. aureus* in biofilm. If there was observed a difference in efficacy between these antiseptics, a second aim was to find the most effective of the three antiseptics.

## Methods

### Bacterial strains and culture conditions

Three wild-type strains of *S. aureus* with Norwegian Veterinary Institute biobank strain identification numbers 1378–1, 300–1 and 132–323 of animal origin were used. The strains were stored at – 70°C in Brain Hearth Infusion broth (BHI; Difco, BD, NJ, USA) supplemented with 15% glycerine (Merck KGaA, Darmstadt, Germany) and recovered on blood agar at 37 ± 1°C overnight (18–24 h).

### Antiseptics

Pyrisept (solution 1 mg/ml cetylpyridinklorid; Weifa, Oslo, Norway), chlorhexidine (chlorhexidine diacetate 1 mg/ml; Fresenius Kabi, Halden, Norway) and iodine (2% liniment NAF: Iodine 2 g/ml, Potassium iodine 1.4 g/ml, ethanol 96%; A/S Den norske Eterfabrikk, Oslo, Norway) were used. The iodine liniment contained 96% ethanol and to test the possible effect of this ethanol on *S. aureus* in biofilm, 96% ethanol (Kemetyl Norge AS, Vestby, Norway) was tested in the biofilm experiment, in addition to the antiseptics.

### Susceptibility test

To verify that the bacterial strains used in the experiment were susceptible for the antiseptics tested, single colonies from a fresh overnight cultures on blood agar were picked and transferred into sterile saline to McFarland 0.5 and the suspension was spread on blood agar using an automated plate spreader. Aliquots of 10 μl of each of the three antiseptics and ethanol were added to separate blank 6 mm paper discs (BD, Sparks, MD, USA) that were placed on top of the agar. Sterile saline was used as a control. Ethanol (96%) was not found possible to evaluate in this system as absolute ethanol is volatile and evaporated quickly from the discs. Agar plates were incubated at 37°C overnight before zone diameters were measured using a ruler. The experiment was performed three times.

### Biofilm experiments

To create an overnight culture in broth, a single colony were picked from the overnight culture on blood agar and transferred into Trypticase Soy Broth (TSB; Oxoid Ltd, Hampshire, England) and incubated statically at 37°C overnight. Biofilms were grown on autoclaved microscope slides (76 x 26 mm, Menzel GmbH + CoKG, Braunschweig, Germany) using overnight culture in TSB supplemented with 1% glucose and 1% NaCl (TSB 1 + 1) in the ratio 1:100 and incubated statically at 37°C for 24 h. After incubation, the biofilm were washed three times in sterile saline to remove loosely adhered bacteria. To simulate a “flushing of wound” procedure, the biofilms were submerged in antiseptic, ethanol or saline solution for 3 sec followed by drying in room temperature for 10 min. Thereafter, the microscope slides were submerged in Dey Engley Neutralizing broth (Difco). Subsequently the microscope slides were washed in saline solution before the biofilms were removed by scraping with a sterile cell scraper (BD Falcon, Bedford, MA, USA) and transferred to sterile reagent tubes containing 5 ml sterile saline and 20 glass beads (3 mm; Assistent, Glaswarenfabrik Karl Hecht GmbH & Co KG, Bavaria, Germany). The tubes were vortexed at 2000 rpm for one minute and the solution was serial diluted in sterile saline, plated onto blood agar and recovered at 37°C for 24 h. After incubation, the number of colony forming units (cfu) was counted. All experiments were performed three times.

### Statistics

Statistical analyses were performed using JMP version 9.0.0 (SAS Institute, Cary, NC, USA) and the non-parametric Wilcoxon/ Kruskal-Wallis test was used to calculate significance. The level of significance was set to *P* < 0.05 in all experiments.

## Results

**Figure 1:**
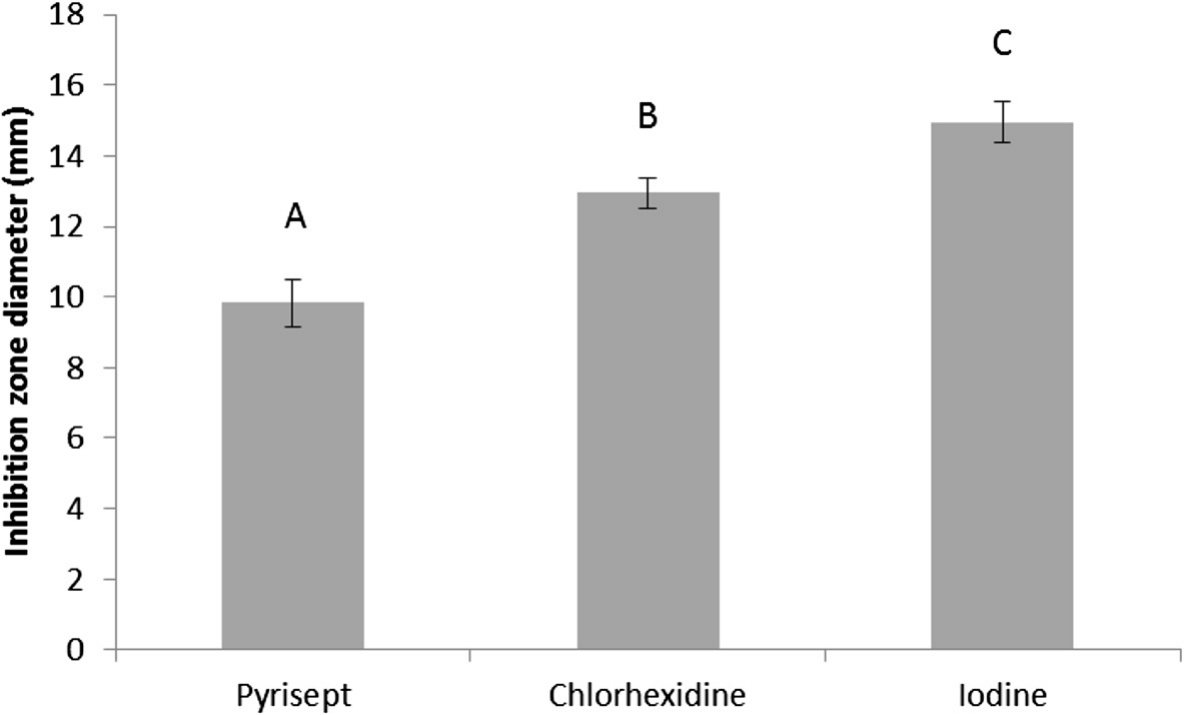
Inhibition zone diameter. Mean inhibition zone diameter in millimeter for each antiseptic used in the experiment. The results are presented with standard deviation. Levels not connected by the same letter are significantly different (*P* <0.05).

Distinct growth inhibition zones were seen for all three antiseptics, indicating that the bacterial strains used were susceptible for all antiseptics used in the experiments. Although all antiseptics showed inhibition zones, significant differences were found in inhibition zones diameter between all antiseptics (*P* = 0.027) (Figure 1).

**Figure 2:**
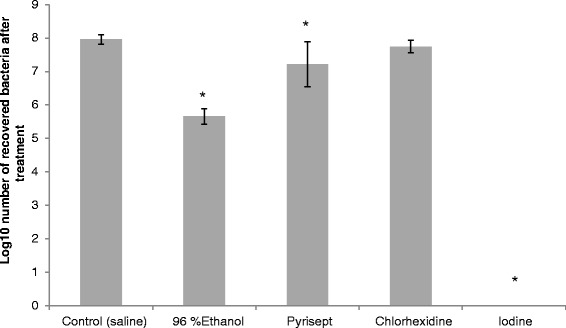
Effect of antiseptics against *Staphylococcus aureus* in biofilm *in vitro*. Bars represent mean log_10_ recovered bacteria after treatment. Results are presented with standards deviation. Iodine treatment gave no recovered bacteria after treatment in any of the experiments. Bars marked with “*” shows treatments with significant reduction compared to the control (*P* <0.05).

Large differences in the bactericidal effect of the different antiseptics against *S. aureus* in biofilm were observed in this study (Figure 2). Iodine treatment was found to be the most effective as no colony forming units (cfu) was detected after treatment (*P* = 0.037). Treatment with sterile saline was used as a control and showed that the average number of cfu in the biofilm was 1.0x10^8^. Exposure to 96% ethanol was the second most effective treatment as the average number of recovered bacteria from the biofilm were reduced by 2.3 log_10_ from the control (*P* = 0.049). Treatment with Pyrisept resulted in a 0.7 log_10_ reduction from the control (*P* = 0.049). Treatment with chlorhexidine was not found significant with 0.2 log_10_ (*P* = 0.126) reduction from the control.

Results showed that the bactericidal effect of 96% ethanol is significantly less than that of 2% iodine liniment containing 96% ethanol (*P* = 0.037).

## Discussion

Striking differences in the bactericidal effect of the different antiseptics against *S. aureus* in biofilm were observed in this study. Interestingly, the observed differences were not fully correlated to differences in bactericidal effect on planktonic bacteria. Chlorhexidine was less effective than Pyrisept in the biofilm experiments, but gave larger inhibition zones of planktonic growth on agar. One likely hypothesis that could explain for the differences in effect against *S. aureus* in biofilm could be that the three different antiseptics have different permeability through the extracellular matrix and/or that the antiseptics contain components that react with the matrix. This could subsequently give large differences in effect against *S. aureus* in biofilm as observed in this study.

Pyrisept contains the active ingredient cetylpyridinum chloride, which is a cationic quaternary ammonium compound (QAC) known for its use as a surface active agent with antibacterial activity by binding to the cell membrane [7,8,10]. Cetylpyridinum chloride is known to be effective against Gram positive bacteria but the effect is reduced in the presence of organic compounds [7]. This may explain the poor efficacy against *S. aureus* in biofilm as biofilm matrix contains organic materials such as proteins [11]. Chlorhexidine, which is a biguanide, showed practically no bactericidal effect on *S. aureus* in biofilm, in the present study. Other studies have found better result using chlorhexidine by prolonging the contact time up to 15 min [9,12]. The antibacterial action of chlorhexidine is by adsorption to the cell membrane [9]. The fact that both cetylpyridinum chloride and chlorhexidine act directly with the bacterial cell membrane and both are reported to have reduced efficacy in the presence of organic material supports our results of being non-efficient against *S. aureus* in biofilm.

Although the exact antimicrobial action of iodine is unknown, it has been suggested that the working action of iodine is that it attacks key groups of proteins, nucleotides and fatty acids which results in cell death [7]. Proteins and nucleotides are key components of the extracellular protective matrix of *S. aureus* [13]. An attack on these matrix components by iodine may result in disruption of the matrix, leaving the bacterial cells less protected against the bactericide. This may explain why it displays a more efficient antiseptic action against biofilm bacteria than cell surface active antiseptics such as QAC’s and chlorhexidine. In addition to iodine compounds, 2% iodine liniment also contains 96% ethanol. The antibacterial effect of ethanol is well known [7] and needed to be tested in this study as it may be the causing or contributing factor to the good results observed for iodine liniment. The use of 96% ethanol in the present study showed that ethanol definitely has a bactericidal effect on *S. aureus* in biofilm but the iodine treatment is far more effective than ethanol alone. Ethanol and ethanol containing disinfectants has previously been shown to be very effective against bacteria in biofilm [14]. The observed effect of ethanol in the present study compared to the study by Moretro *et al*. [14] is far less and reasons for this might be that ethanol is more effective against *Salmonella* than *S. aureus,* in general or when in biofilm. The contact time is longer in the study by Moretro *et al.* [14] than in the present study and can also explain the conflicting results. Interestingly, the antibacterial effect of ethanol has been shown to be almost identical to iodine by denaturing proteins [7]. For this reason, it is likely that both iodine and ethanol together contribute to the exceptionally good results obtained by 2% iodine liniment in this study. The exact mode of action of 2% iodine against *S. aureus* in biofilm should be explored further as this is important knowledge with regards to selecting the antiseptics that are most likely to be successful in treating infected wounds harboring biofilms.

In our study, we submerged the pre-formed biofilms in antiseptics for 3 sec and then the biofilms were removed from the antiseptic solution and dried in room temperature for 10 minutes. This was to simulate a “flushing of a wound” procedure. It is recommended that a modular approach be taken to first test the formulation *in vitro*, then proceed to *in vivo* testing if the *in vitro* testing is successful. In this *in vitro* model, we used sterile microscope slides as a basis for *S. aureus* to form biofilm. The reason for selecting these conditions was to study the isolated effect of the antiseptics on bacteria in biofilm. *In vivo* in wounds, a large number of additional factors will be present that may influence the effects of the antiseptics. However, if an antiseptic has little or no effect in our system, it will most likely be even less effective under the more challenging conditions *in vivo*.

## Conclusions

The bactericidal effect of the different antiseptics used in the present study was found to vary significantly against *S. aureus* in biofilm. Treatment with 2% iodine liniment was found to eradicate *S. aureus* in biofilm *in vitro* whilst chlorhexidine and Pyrisept showed less bactericidal effect. For this reason, the present study indicates that iodine might be the best choice of the three antiseptics tested for use against *S. aureus* in biofilm. The present study also contributes to focus attention on the importance of qualifying antiseptics based on results using biofilm bacteria rather than planktonic bacteria.

## References

[1] Costerton JW, Stewart PS, Greenberg EP (1999). Bacterial biofilms: a common cause of persistent infections. Science.

[2] Percival SL, Hill KE, Williams DW, Hooper SJ, Thomas DW, Costerton JW (2012). A review of the scientific evidence for biofilms in wounds. Wound Repair Regen.

[3] Cooper R (2010). Biofilms and wounds: much ado about nothing?. Wounds UK.

[4] Brackman G, De Meyer L, Nelis HJ, Coenye T (2013). Biofilm inhibitory and eradicating activity of wound care products against *Staphylococcus aureus* and *Staphylococcus epidermidis* biofilms in an *in vitro* chronic wound model. J Appl Microbiol.

[5] Diekema DJ, Pfaller MA, Schmitz FJ, Smayevsky J, Bell J, Jones RN (2001). Survey of infections due to *Staphylococcus species*: frequency of occurrence and antimicrobial susceptibility of isolates collected in the United States, Canada, Latin America, Europe, and the Western Pacific region for the SENTRY Antimicrobial Surveillance Program, 1997–1999. Clin Infect Dis.

[6] Fridkin SK, Hageman JC, Morrison M, Sanza LT, Como-Sabetti K, Jernigan JA (2005). Methicillin-resistant *Staphylococcus aureus* disease in three communities. New Engl J Med.

[7] McDonnell G, Russell AD (1999). Antiseptics and disinfectants: activity, action, and resistance. Clin Microbiol Rev.

[8] Gilbert P, Moore LE (2005). Cationic antiseptics: diversity of action under a common epithet. J Appl Microbiol.

[9] Bonez PC, Dos Santos Alves CF, Dalmolin TV, Agertt VA, Mizdal CR, Flores Vda C (2013). Chlorhexidine activity against bacterial biofilms. Am J Infect Control.

[10] Masadeh MM, Gharaibeh SF, Alzoubi KH, Al-Azzam SI, Obeidat WM (2013). Antimicrobial activity of common mouthwash solutions on multidrug-resistance bacterial biofilms. J Clin Med Res.

[11] Otto M (2008). Staphylococcal biofilms. Currt Top Microbiol.

[12] Tote K, Horemans T, Vanden Berghe D, Maes L, Cos P (2010). Inhibitory effect of biocides on the viable masses and matrices of *Staphylococcus aureus* and *Pseudomonas aeruginosa* biofilms. Appl Environ Microbiol.

[13] Kiedrowski MR, Horswill AR (2011). New approaches for treating staphylococcal biofilm infections. Ann NY Acad Sci.

[14] Moretro T, Vestby LK, Nesse LL, Storheim S, Kotlarz K, Langsrud S (2009). Evaluation of efficacy of disinfectants against Salmonella from the feed industry. J Appl Microbiol.

